# Sepsis Prediction: Biomarkers Combined in a Bayesian Approach

**DOI:** 10.3390/ijms26157379

**Published:** 2025-07-30

**Authors:** João V. B. Cabral, Maria M. B. M. da Silveira, Wilma T. F. Vasconcelos, Amanda T. Xavier, Fábio H. P. C. de Oliveira, Thaysa M. G. A. L. de Menezes, Keylla T. F. Barbosa, Thaisa R. Figueiredo, Jabiael C. da Silva Filho, Tamara Silva, Leuridan C. Torres, Dário C. Sobral Filho, Dinaldo C. de Oliveira

**Affiliations:** 1 Postgraduate Program in Therapeutic Innovation, Federal University of Pernambuco-UFPE, Professor Moraes Rego Avenue, SN, University City, Recife 50670-420, Brazil; dinaldo@cardiol.br; 2 Postgraduate Program in Nursing, Federal University of Paraíba-UFPB, João Pessoa 58051-900, Brazil; wilmafreire23@gmail.com; 3 College of Nursing, University of Pernambuco-UPE, Recife 50670-901, Brazil; mariana.barrosmelo@upe.br (M.M.B.M.d.S.); thaisa.remigiofigueiredo@upe.br (T.R.F.); jabiael.filho@upe.br (J.C.d.S.F.); tamarasilva2@gmail.com (T.S.); 4 Academic Center of Vitória, Federal University of Pernambuco-UFPE, Recife 50670-420, Brazil; amanda.txavier@ufpe.br; 5 Department of Biostatistics, Federal Rural University of Pernambuco-UFRPE, Recife 52171-030, Brazil; fportella@gmail.com; 6 Institute of Integral Medicine Professor Fernando Figueira-IMIP, Recife 50070-550, Brazil; thaysamgal@gmail.com (T.M.G.A.L.d.M.); leuridan.torres@gmail.com (L.C.T.); 7 College of Nursing, State University of Paraíba-UEPB, Campina Grande 58429-500, Brazil; keylla.fernandes@servidor.uepb.edu.br; 8 Postgraduate Program in Health Sciences, University of Pernambuco-UPE, Recife 50670-420, Brazil; dsobral@cardiol.br

**Keywords:** sepsis, biomarkers, Bayes theorem, sTREM-1, cardiac surgery

## Abstract

Sepsis is a serious public health problem. sTREM-1 is a marker of inflammatory and infectious processes that has the potential to become a useful tool for predicting the evolution of sepsis. A prediction model for sepsis was constructed by combining sTREM-1, CRP, and a leukogram via a Bayesian network. A translational study carried out with 32 children with congenital heart disease who had undergone surgical correction at a public referral hospital in Northeast Brazil. In the postoperative period, the mean value of sTREM-1 was greater among patients diagnosed with sepsis than among those not diagnosed with sepsis (394.58 pg/mL versus 239.93 pg/mL, *p* < 0.001). Analysis of the ROC curve for sTREM-1 and sepsis revealed that the area under the curve was 0.761, with a 95% CI (0.587–0.935) and *p* = 0.013. With the Bayesian model, we found that a 100% probability of sepsis was related to postoperative blood concentrations of CRP above 71 mg/dL, a leukogram above 14,000 cells/μL, and sTREM-1 concentrations above the cutoff point (283.53 pg/mL). The proposed model using the Bayesian network approach with the combination of CRP, leukocyte count, and postoperative sTREM-1 showed promise for the diagnosis of sepsis.

## 1. Introduction

Sepsis is recognized as a clinical syndrome characterized by biological, physiological, and biochemical alterations, culminating in organ dysfunction secondary to exacerbated inflammatory responses to an infection. It is a continuum of severity, starting with an uncomplicated infection, which can evolve into sepsis and then septic shock, which can culminate in a syndrome of multiple organ dysfunction and, ultimately, death [1,2].

The World Health Organization (WHO), on the basis of data compiled up to 2015, noted that sepsis caused approximately 5.9 million deaths in children under 5 years of age. Most of these deaths occur in developing countries and are associated with infectious diseases, in which the term “severe” is used to translate conditions of poor tissue perfusion, acidosis, and hypotension, the classic hallmarks of sepsis, and septic shock. The incidence of sepsis in Latin America, especially in pediatrics, is limited, but the mortality rate is high, ranging from 25% to 67%. Socioeconomic factors can influence both the occurrence and outcomes of sepsis since inequalities in the availability of and access to health services result in worse outcomes for patients with sepsis admitted to public hospitals in developing countries [3,4].

Therefore, a strategy for the early diagnosis of sepsis is the measurement of biomarkers, which are characterized as substances that indicate normal biological processes, as well as pathological processes. In this context, soluble triggering receptor expressed on myeloid cells-1 (sTREM-1), a transmembrane protein discovered by Swiss researchers in 2000 and a member of the immunoglobulin family expressed on the surface of neutrophils, monocytes, and macrophages, plays a regulatory role in signaling pathways [5] and has been studied as a biomarker of inflammatory and infectious states, and its use has shown good results [6,7,8].

In addition, during sepsis, the cellular and humoral immune response is activated, resulting in hematological and immunological alterations. Leukocytosis, especially an increase in granulocytes and neutrophils, leads to an increase in the overall count, which is usually accompanied by a leftward shift and the presence of immature cells. C-reactive protein (CRP) is another widely used marker, especially for monitoring inflammatory processes, both of which are typically used in the evaluation of septic patients [9].

Over the last few years, artificial intelligence (AI) tools have been increasingly used in the health sector and are considered highly effective methods, especially for developing predictive models and identifying important factors for diagnosing diseases and guiding therapeutic models [10]. Bayesian networks (BNs), which are based on Bayes’ theorem, are an example of this type of tool. They can be defined as a probabilistic network, represented by a graphical structure formed by relationships between the nodes and their parameters [11]. The sensitivity and specificity of clinical signs of sepsis and commonly used laboratory tests are not considered satisfactory in the diagnosis of sepsis, and their individual use is not indicated. Furthermore, their combined evaluation is often subjectively influenced by healthcare professionals. Current criteria for sepsis diagnosis, which are based on markers such as CRP and leukocyte counts, have significant limitations. The low sensitivity and specificity of these methods hinder the early detection of dysregulated inflammatory responses, particularly in the initial stages. Signs such as leukocytosis may be absent in immunocompromised patients or conflated with other conditions, whereas CRP cannot reliably distinguish between bacterial, viral, or noninfectious processes. Moreover, traditional clinical assessment is subjective and relies on the interpretation of nonspecific symptoms, which delays diagnosis and worsens outcomes, especially in resource-limited regions. The pursuit of new biomarkers, such as sTREM-1, is justified by their ability to identify pathogen-specific immune responses, enhancing diagnostic precision. Integrating multiple parameters (sTREM-1, CRP, and leukocytes) via a BN has emerged as an innovative strategy, combining data objectively and reducing subjective variability. This approach enables the identification of complex patterns and risk stratification, optimizing early detection and clinical guidance—key factors in preventing progression to septic shock and mortality [12]. From this perspective, IA has emerged as an intelligent structure that allows not only the combination of isolated parameters but also their use on the basis of stratification and adjusted cutoff points, ensuring greater safety and objectivity for the diagnosis and management of the disease.

Therefore, we aimed to build a prediction model for sepsis by combining sTREM-1, CRP, and leukocyte counts through a BN. We hypothesized that combining these biomarkers through the BN could be useful for sepsis detection.

## 2. Results

Among the 32 patients, most were male (56.25%), born at term (93.75%), and underwent elective surgery (53.12%). The mean age of the patients was 2 years (±3.0), with a height of 75.6 cm (±25.3) and weight of 9.9 kg (±8.4). Sepsis was present in 41% of the patients. Postoperative sTREM-1 values ranged from 113.70 pg/mL to 904.04 pg/mL, with a mean of 302.77 pg/mL (±DP 171.12); CRP ranged from 4.9 mg/dL to 208.7 mg/dL, with a mean of 63.28 mg/dL (±DP 63.25); and the WBC count ranged from 6280 cells/µL to 44,792 cells/µL, with a mean of 17.713 cells/µL (±DP 10.573). We found that during the postoperative period, the mean sTREM-1 level was greater among patients diagnosed with sepsis than among those not diagnosed with sepsis (sTREM-1 post = 394.58 pg/mL (±DP 211.91) versus 239.93 pg/mL, (±DP 100.88) *p* < 0.001). Analysis of the Receiver Operating Characteristic Curve (ROC) curve for sTREM-1 and sepsis revealed that the area under the curve (AUROC) was 0.761, with a 95% CI (0.587–0.935) and *p* = 0.013, as shown in Table 1.

**Figure 1:**
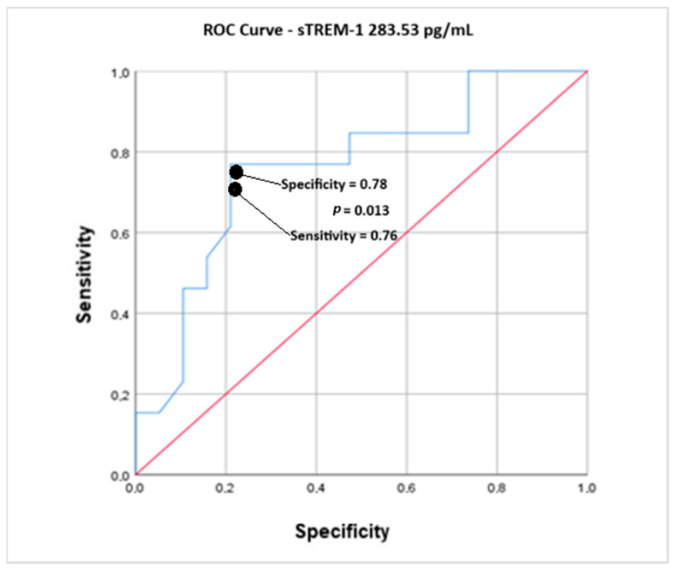
ROC curve analysis of postoperative sTREM-1 expression versus the diagnosis of sepsis in children undergoing cardiac surgery (*n* = 32).

According to the data shown in Figure 1 and Table 2, the cutoff point for diagnosing sepsis via sTREM-1 was 283.53 pg/mL, with a sensitivity of 0.769 and specificity of 0.789 (J = 0.56, positive likelihood ratio (PVR) = 3.64455 and negative likelihood ratio (NLR) = 0.292776).

**Figure 2:**
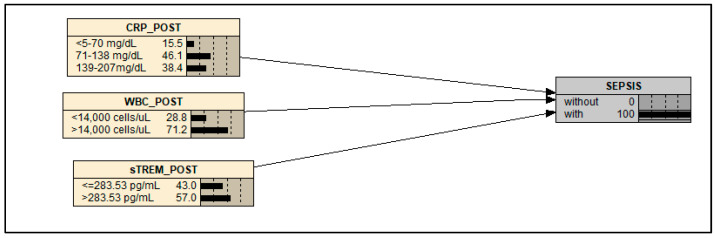
Bayesian network model structure showing the relationships between postoperative CRP levels, WBC counts, and sTREM-1 levels and the diagnosis of sepsis in children undergoing cardiac surgery (*n* = 32).

We found a 100% probability of sepsis related to postoperative blood concentrations of CRP above 71 mg/dL, WBC count values above 14,000 cells/μL, and sTREM-1 concentrations above the cutoff point (283.53 pg/mL), as shown in Figure 2.

## 3. Discussion

Sepsis is one of the main causes of hospitalization in pediatric patients. Recently, a series of initiatives have been set out not only to improve understanding and determine better concepts for sepsis but also to reduce morbidity and mortality and subsequent impacts through timely diagnosis and the establishment of early therapy guided by specific guidelines for the pediatric population. In Brazil, the prevalence of sepsis in pediatric intensive care settings is estimated to be 25.0%, with approximately 74.6 cases per 100.000 pediatric population (95% CI 61.5–90.5), which translates into 42.374 cases per year (34.940–51.443), with an estimated mortality of 8.305 (6.848–10.083) [2].

In this context, machine-learning models are emerging as promising tools for the detection and management of sepsis in intensive care units (ICUs). These models use complex algorithms and statistical methods to learn from patient data, including vital signs and laboratory tests, to predict the onset of sepsis. Early identification and treatment of sepsis are associated with improved outcomes, so machine-learning-based alert systems can reduce the time to recognition. Using these models to predict the onset of sepsis in the ICU has the potential to revolutionize the way the disease is detected, treated, and managed, leading to better outcomes for patients and reduced healthcare costs [13,14].

In the context of cardiac surgery, patients who undergo cardiopulmonary bypass can experience exacerbated inflammation and postoperative infections, which can culminate in serious complications, especially those associated with systemic organ dysfunction. Although early diagnosis is essential, a single specific biomarker to detect hyperinflammatory states or organ dysfunction has not yet been established in clinical practice [15].

Among the inflammatory markers used to identify possible alterations is CRP, which is an inflammatory protein synthesized in hepatocytes. The blood concentration of CRP increases rapidly, by approximately 25%, during inflammatory states. During the postoperative period, these methods are more sensitive for detecting complications than elevations in leukocytes, heart rate, or the appearance of fever [16,17].

A study carried out by Zeng et al. (2022) [18], with 129 children divided into four groups (non-septic infection, hyperinflammatory disease, organ dysfunction, and control groups), revealed that the CRP level (67.08 [1.00~175.40] mg/L) was significantly greater in the infected group than in the noninfected group. The marker’s potential to distinguish children in a state of hyperinflammatory disease from those with infection without sepsis was also assessed, and on the basis of clinical data, the CRP cutoff point was set at 25.11 mg/L, with a sensitivity rate of 86.57% and specificity of 96.97%.

Although CRP has been extensively studied as a diagnostic marker, Lanzioti et al. (2016) [19] noted that, owing to its limited specificity, the combined use of CRP with other biomarkers has been tested. Importantly, as it is not a specific biomarker for distinguishing infection from inflammation or for distinguishing specific infectious agents, its use, as with other biomarkers, should always be associated with the clinical assessment of the patient and the use of clinical criteria for decision-making. Its use in combination with other biomarkers, when available, is promising for increasing its specificity in diagnosing infections. Notably, the use of biomarkers, both for diagnosis and for predicting the outcome of sepsis, is described in the literature, but owing to their nonspecific nature and insufficient predictive value for individuals, research is still focusing on new aspects of biomarkers related to sepsis [20]. Among them, sTREM-1 stands out, since infectious and inflammatory stimuli can lead to its elevation, making it a valuable diagnostic parameter for monitoring macrophage activation under inflammatory conditions. Barati et al. (2010) [20] reported that in ICU patients, sTREM-1 and CRP concentrations were higher in the sepsis group than in the systemic inflammatory response syndrome (SIRS) group (*p* < 0.001), which was also observed by Su et al. (2012) [8], who reported that the sepsis group had higher serum levels of sTREM-1 and CRP than did the SIRS group (*p* < 0.05). In addition, a meta-analysis conducted by Wu et al. (2012) [21] revealed that plasma sTREM-1 had a moderate diagnostic performance in differentiating sepsis from SIRS.

The AUROCs for these indicators were 0.868 (95% CI 0.798–0.938) and 0.679 (95% CI 0.578–0.771), respectively. With 108.9 pg/mL as the cutoff point for sTREM-1, the sensitivity was 0.83, and the specificity was 0.81. Stein et al. (2014) [22] reported that the performance of CRP and sTREM-1 for sepsis has a sensitivity of 45% and 82%, respectively, and that the specificity of CRP and sTREM-1 is 82% and 48%, respectively, which suggests that their use in combination may present better results in the diagnosis of sepsis [6]. In addition, Caldas et al. (2008) [23] analysed the combined performance of the WBC count and CRP and determined that the latter was significantly better than its use alone. The combination of these tests increased the diagnostic accuracy of the combination of the WBC count and CRP level, proving useful in the diagnosis of sepsis and comparable to the use of IL-6 and TNF-α. Therefore, as both are routine tests in intensive care units, the correct interpretation and use of these tests are highly valuable.Research has shown that measuring sTREM-1 may also be useful in predicting the severity of inflammatory diseases, especially when there is an inadequate immune response, as occurs in patients with sepsis [24]. The authors also demonstrated that patients with septic shock have extremely high levels of sTREM-1 compared with healthy individuals, where sTREM-1 was detectable in only 19% of individuals. Furthermore, elevated sTREM-1 levels have been associated with scales that predict sepsis severity, such as the Sequential Organ Failure Assessment (SOFA) and the Acute Physiology and Chronic Health Evaluation (APACHE II) [25,26,27]. In our study, the sTREM-1 cut-off for the diagnosis of sepsis was 283.53 pg/mL, a value close to that reported by Petric et al. (2018) [27], who reported that 300 pg/mL was the optimal value for the diagnosis of septic patients (*p* = 0.021). A meta-analysis that included patients with neonatal sepsis revealed that the pooled sensitivity and specificity of sTREM-1 in the diagnosis of sepsis were 0.94 (95% CI 0.82–0.98) and 0.87 (95% CI 0.70–0.95), respectively, which demonstrates that the biomarker in question has high sensitivity and specificity for the diagnosis of sepsis [6]. Notably, the combination of biomarkers is a hot topic in clinical practice, as it allows for better patient management, especially in distinguishing between inflammatory and infectious processes, aiding in decision-making regarding the use of antibiotic therapy, for example. Therefore, the measurement of these biomarkers and their combination through a BN has potential application value in the detection of patients with sepsis.Among the positive aspects, we highlight the fact that the model demonstrated the use of BN, an easy-to-use AI tool that combines CRP levels and leukocyte counts—widely used, low-cost, and easily obtainable parameters—and sTREM-1, a new biomarker with excellent potential for identifying sepsis.Although this study provides relevant contributions, several limitations should be acknowledged. As a cross-sectional analysis without randomization, causal relationships between the analyzed variables cannot be definitively established. Furthermore, the focus on children undergoing cardiac surgery inherently limits the generalizability of our findings, as the results reflect the specific characteristics of this patient population. The model requires validation in larger cohorts, followed by implementation studies to assess its clinical utility and real-world impact.The relatively small sample size, while justified by the specialized clinical context, may compromise the statistical robustness of the analyses and the external validity of the conclusions. This limitation also increases the risk of model overfitting, which could artificially inflate the predictive performance to the detriment of generalizability. Furthermore, although the proposed model integrates three biomarkers (sTREM-1, CRP, and leukocyte count), it does not consider multivariate interactions or identify dominant predictors among these variables. The absence of statistical interaction terms limits the ability to detect potential synergistic or antagonistic effects between biomarkers, which could increase diagnostic accuracy. To address these gaps, complementary analyses—such as logistic regression models incorporating interaction terms—are recommended to assess whether specific combinations of sTREM-1, CRP, and leukocyte counts disproportionately influence predictive power. Therefore, these findings should be interpreted with caution until they are replicated in larger, more diverse cohorts via standardized protocols.

## 4. Materials and Methods

This is an exploratory translational study. The study was carried out with 32 children between the ages of 0 and 11 with congenital heart disease who had undergone surgical correction at a public referral hospital in northeast Brazil, which provides care for individuals with cardiovascular diseases in various life cycles through the Brazilian Unified Health System (SUS).

The study included individuals of both sexes with a medical diagnosis of congenital heart disease with an indication for partial or total cardiac surgical correction. Children with rare genetic syndromes, those with an infectious diagnosis on admission, or those who had already undergone palliative or total correction surgery before the data collection period were excluded. The population consisted of all the children with congenital heart disease seen at the health service over a 17-month period, which totaled 45 patients. This number represents the total number of surgeries that occurred in the service, within the period stipulated for the study. However, after the inclusion and exclusion criteria were defined, as well as the consent of family members, the sample consisted of 32 children.

Data were collected between June 2021 and November 2022 via a structured instrument with sociodemographic variables (age, sex, color, city of residence, and state of residence) and clinical variables (type of surgery, surgical complications, presence of cyanosis, and laboratory tests), and the determination of sepsis was made based on the presence of this diagnosis in the patient’s medical records within 48 h after surgery, through the records of the responsible medical team, in accordance with the following criteria according to the age group: heart rate, respiratory rate, temperature, systolic blood pressure and number of leukocytes and/or change in perfusion (capillary filling time > 2 s), acute change in neurological status, desaturation (<92% in room air), low urinary flow (≤0.5 mL/kg/h), and global hypotension according to age.

To determine sTREM-1, blood samples were taken during the immediate preoperative period and immediately after surgery. Blood samples of 1.0 mL were collected. The samples were then centrifuged to obtain serum and plasma and stored at −80.0 °C. To measure sTREM-1, the enzyme-linked immunosorbent assay (ELISA) method was used with a commercial Human TREM-1 ELISA Kit (DuoSet^®^ ELISA Development System, R&D Systems^®^, a biotechne^®^ Brand, Minneapolis, MN, USA) according to the manufacturer’s instructions. A well-washed microplate reader (Thermo Fisher Scientific Oy., Vantaa, Finland) was used. CRP and leukocyte values were obtained from the patient’s medical records.

To determine the value of the sTREM-1 cutoff point after cardiac surgery and relate it to sepsis (occurrence or not), an ROC curve was constructed, with a significant robustness of at least 70% of the area and *p* < 0.05. The best cutoff point was determined via the Youden test (J). The probability of sepsis occurring for sTREM-1 concentrations above the cutoff point determined by the J index was determined via the positive likelihood ratio (PLR). On the other hand, the probability of a false negative for a given outcome in patients with sTREM-1 concentrations above the cutoff point was assessed via the negative likelihood ratio (NLR). All statistical analyses were carried out via IBM SPSS Statistics^®^ software version 26.0, as it was suitable for achieving the study’s objectives and for enabling the accuracy and generalization of its results.

For the construction of the BN model, the following steps were implemented: (1) definition of the model’s objectives and its target (outcome) node; (2) generation of a model structure, including nodes and directed edges (arrows), based on the variables studied in this manuscript; (3) quantification of probabilistic relationships between variables [28]. The BN model was developed using Netica version 6.09^®^ software (https://www.norsys.com (accessed on 3 January 2023)).

In a BN model, each node (variable) is typically defined by a probability distribution across discrete intervals or categorical states. The network structure integrates these distributions into a Conditional Probability Table (CPT). Nodes from which edges originate are termed parent nodes, whereas nodes receiving edges are termed child nodes. A CPT defines the probabilistic dependency between child nodes and their parent(s). During model execution, the probability distribution of child nodes is updated according to Bayes’ theorem [28].

The probability distributions within the CPT account for natural variability in the data as well as the uncertainty inherent to the relationships between variables. The complexity of a BN increases exponentially with the number of nodes and edges [13]. Consequently, this study incorporated variables rigorously associated with postoperative sepsis episodes: CRP levels, postoperative leukocyte counts, and post-surgical sTREM-1 concentrations. The model aims to predict the probability of sepsis occurrence for each indicator.

Continuous variables were discretized into intervals for use as discrete nodes in the BN. The number of states per variable was minimized (Table 3) to reduce model complexity as the number of variables increased, while preserving the method’s sensitivity in assessing sepsis probability.

The CPT for each child node was derived from the frequency distribution of its parent node variables. Each column within a CPT represents the probability distribution of the child node’s states for a specific combination of its parent nodes’ states. The complete table accounts for three strata of the CRP, two strata of the postoperative WBC, and two strata of the sTREM-1 concentrations. This yields a total of 12 columns (3 × 2 × 2), reflecting all possible strata combinations (Table 4).

## 5. Conclusions

This study demonstrates the potential diagnostic utility of a Bayesian network model integrating soluble myeloid trigger receptor-1, C-reactive protein, and leukocyte count to identify postoperative sepsis in pediatric patients. The main findings revealed significantly elevated postoperative sTREM-1 levels in septic patients compared with non-septic patients, establishing sTREM-1 as a discriminative biomarker. ROC curve analysis demonstrated its diagnostic capability, with an optimized cutoff threshold. Notably, the synergistic application of sTREM-1, CRP, and leukocytosis achieved a 100% predictive probability for sepsis, highlighting the additive value of multiparameter analysis. While these results highlight the feasibility of AI-based models to improve diagnostic accuracy, the limited sample size and homogeneity of the studies require external validation in larger and more diverse cohorts. Future research should explore the temporal dynamics of biomarkers, their cost-effectiveness, and their integration into clinical workflows to assess their translational impact. However, this proof-of-concept framework provides a compelling basis for leveraging novel biomarkers alongside conventional inflammatory indices to address the urgent need for early sepsis detection of sepsis in high-risk surgical populations.

## Figures and Tables

**Table 1:** Area under the postoperative sTREM-1 curve versus diagnosis of sepsis in children undergoing cardiac surgery (*n* = 32).

Area Under the Curve			
Test Outcome Variables: Postoperative sTREM-1 and Sepsis			
Area	*p* Value	Confidence Interval 95%	
		Lower Limit	Upper Limit
0.761	0.013	0.587	0.935
AUROC 0.761 95% CI—0.587–0.935/*p* = 0.013			

Legend: AUROC = Area under the ROC curve.

**Table 2:** Postoperative sTREM-1 cutoff points, sensitivity, and specificity versus diagnosis of sepsis in children undergoing cardiac surgery (*n* = 32).

Curve Coordinates					
Test Outcome Variables: Postoperative sTREM-1 and Sepsis					
sTREM-1 Cutoff Points—Postoperative	Sensitivity	Specificity	J	PLR	NLR
112.70	1.000	0.000	0.00		
122.48	1.000	0.053	0.05		
139.00	1.000	0.105	0.10		
155.40	1.000	0.158	0.16		
167.44	1.000	0.263	0.26		
177.76	0.923	0.263	0.18		
188.91	0.846	0.263	0.11		
195.29	0.846	0.316	0.16		
199.58	0.846	0.368	0.21		
204.64	0.846	0.421	0.27		
208.26	0.846	0.474	0.32		
214.89	0.846	0.526	0.37		
221.54	0.769	0.526	0.29		
228.32	0.769	0.579	0.35		
238.96	0.769	0.684	0.45		
262.72	0.769	0.737	0.50		
**283.50**	**0.769**	**0.787**	**0.56**	**3.64**	0.292 *
286.84	0.692	0.789	0.48		
297.79	0.615	0.789	0.40		
335.73	0.538	0.842	0.38		
368.14	0.462	0.842	0.30		
381.51	0.462	0.895	0.36		
397.00	0.385	0.895	0.28		
408.88	0.308	0.895	0.20		
434.57	0.231	0.895	0.12		
467.30	0.154	0.470	0.37		
610.79	0.154	1.000	0.15		
822.14	0.077	1.000	0.07		
904.04	0.000	1.000	0.00		

Legend: J = Youden’s test; PLR = positive likelihood ratio; NLR = negative likelihood ratio; * Bold highlighting is based on sTREM-1 cutoff value.

**Table 3:** Description of the nodes of the Bayesian network model generated in this manuscript.

Model Nodes	Unit	Stratification of the Node	
		1	2
CRP_POST	mg/dL	<5–70	139–207
WBC_POST	cells/µL	<14,000	>14,000
sTREM-1_POST	pg/mL	≤283.53	>283.53
SEPSIS	-	Without	With

**Table 4:** Conditional Probability Table for each stratum of BN nodes.

CRP_Post	<5–70		>71	
WBC_Post	<14,000		>14,000	
sTREM_Post	With Sepsis	Without Sepsis	With Sepsis	Without Sepsis
≤283.53	0%	100%	67%	33%
>283.53	20%	80%	100%	0%

## Data Availability

We declare that our primary data is available for consultation upon justified request.

## Abbreviations

The following abbreviations are used in this manuscript:

AI	Artificial Intelligence
APACHE II	Acute Physiology and Chronic Health Evaluation
AUROC	Area under the curve
BN	Bayesian network
CAAE	Certificate of Presentation of Ethical Appreciation
CRP	C-reactive protein
ELISA	Enzyme-linked immunosorbent assay
ICU	Intensive care unit
J	Youden’s test
NLR	Negative likelihood ratio
PLR	Positive likelihood ratio
SIRS	Systemic inflammatory response syndrome
SOFA	Sequential Organ Failure Assessment
SUS	Unified Health System
sTREM-1	Soluble triggering receptor expressed on myeloid cells-1
WBC	White blood cell
WHO	World Health Organization
